# Deep Learning for Parkinson’s Disease Diagnosis: A Graph Neural Network (GNN) Based Classification Approach with Graph Wavelet Transform (GWT) Using Protein–Peptide Datasets

**DOI:** 10.3390/diagnostics14192181

**Published:** 2024-09-29

**Authors:** Prabhavathy Mohanraj, Valliappan Raman, Saveeth Ramanathan

**Affiliations:** 1 Department of Artificial Intelligence and Data Science, Coimbatore Institute of Technology, Coimbatore 641014, India; valliappan@cit.edu.in; 2 Department of Computer Science and Engineering, Coimbatore Institute of Technology, Coimbatore 641014, India; saveeth.r@cit.edu.in

**Keywords:** Parkinson prediction, graph neural network, graph wavelet transform, MDS-UPDRS III scale, Protein–Peptide

## Abstract

**Background:** An important neurological disorder of Parkinson’s Disease (PD) is characterized by motor and non-motor activity of the patients. Empirical condition of the patient: PD assessment uses the Movement Disorder Society Unified Parkinson’s Rating Scale part III (MDS-UPDRS-III) measures for identifying the prediction of PD. Due to the unstable value of the measurement, the PD prediction and tracking lead to a lower prediction rate. **Methods:** To overcome this limitation, this paper proposed the Graph Wavelet Transform (GWT) based weighted feature extraction along with the Graph Neutral Network (GNN) classification. The main contribution of this research is (i) The weighted correlation between the data is calculated by GWT for effective prediction of PD. (ii) Machine learning algorithms were trained to predict Parkinson’s disease based on these patterns. In this research, we developed a new model called Graph Neural Network (GNN) to predict PD tremors’ MDS-UPDRS-III score using input data. To strengthen PD research and enable the construction of individualized treatment plans, these linked networks work together to methodically examine the data and find significant discoveries. **Results:** The proposed approach for predicting PD severity (motor- and MDS_UPDRS) has a mean squared error of 0.1796 and a root mean squared error of 0.2845, according to the experimental data. The prediction accuracy is increased by 27.66%, 54.11%, and 0.71%, correspondingly, when compared with the most effective State-of-the-Art methods of DNN, ANFIS + SVR, and Mixed MLP models. **Conclusion:** In conclusion, this proves that the proposed strategy is more effective at making predictions.

## 1. Introduction

PD is a neurological disease categorized by persistent degeneration. Nobody knows what causes Parkinson’s disease. However, studies have shown that environmental and genetic variables cause PD [1]. To provide a basic understanding, PD is considered to be a problem of the central nervous system [2] This condition results from the death of cells from different brain areas. Members of this cell type also include dopamine-producing neural cells. To coordinate movement, dopamine is crucial. It carries messages from one part of the brain to another using chemical messengers. Patients have mobility problems as a result of these cells being lost [3,4,5].

To properly manage PD, it is essential to evaluate the severity of symptoms and the disease’s development [6,7]. The Unified Parkinson’s Disease Rating Scale (UPDRS) [8] is a tool that medical professionals often use to rate symptoms. The motor symptoms of PD are most often measured by the gold standard of Part III of the MDS-UPDRS assessment instrument. There are a total of eighteen items that assess static balance, bradykinesia, tremor, speech, posture, and gait. On a 5-point scale, we grade each thing, and we give each side of the body its score. No symptoms at all (0), mild impairment (2), moderate impairment (3), and severe impairment (4 on the scale) are the corresponding values. If the patients have symptoms like tremors, then they are advised to complete the assessment test of movement and activity for the measurement of the MDS-UPDRS III scorecard. While the measurement scale has a standard metric value for the prediction category, the predicted value is different when changing the patient. This variability will reduce the reliability of the measurement [9]. Also, this qualitative approach requires more time and resources to measure each symptom score [10].

Proteins and peptides need to connect in many biological processes. Because they have small interface areas and are not toxic, peptides are perfect subjects for therapeutic methods, rational drug design, and inhibiting proteins. A collection of protein-peptide complexes called the PP Dataset may help us predict how proteins distinguish from each other [11,12]. Through organized data analysis, the primary objective of time series data mining is to find new and interesting trends in information about patients. Time series data are used by researchers and medical professionals for various purposes, such as identifying clusters, patterns, and standards, making predictions, and grouping data into classes. Mathematics-based Graph Wavelet Transform (GWT) is useful for processing time series data and can be used for tasks like noise filtering, minimizing data, and finding outliers. The wavelet transform uses attenuation basis functions and limited lengths to provide structurally beneficial results. Information in the spatial and spectral domains may be localized with relative ease using this method. The sparsity of the basis and its inverse in the wavelet transform is another important feature that makes it efficient and useful.

Neuronal networks (NNs) are widely used in healthcare for tasks such as identifying diseases, developing new medicines, and planning individualized treatments [12]. NNs are based on the way the brain works and learn patterns from data [13]. They could be used in Parkinson’s disease studies to predict how the disease becomes more serious, classify stages, and find early warning signs [14,15]. GNN is better than regular neural networks (NNs) at describing data that have been structured in a graph [16]. Although NNs are good at understanding complicated graphs by using nodes and edges relationships, they are not equally effective at understanding sequence data. They work best in areas like recommendation systems, social networks, and medical healthcare research that easily use graph-based data [17,18]. In the medical field, GNNs are useful for many things in addition to the PD domain. They can be used to study gene expression, diagnose conditions, find new drugs, and analyze data from images and datasets. They have created an effective technique for analyzing complex medical data using graphs, which has helped improve our understanding of disease and its treatment. Recently, the relevant study showed a GNN-based way of predicting how neurological diseases like Parkinson’s will progress by examining MRI data of brain-related networks [19]. This GNN model can find changes in the way brain cells are added, which may help physicians predict how the disease will become worse, even in the early stages of Parkinson’s disease. This means it could be a useful tool to identify challenges early and come up with better ways to treat them [20]. Based on a similar study, PD is a widespread neurological disease that a lot of people have. Some types of neurons have this disease due to a combination of genetic and environmental factors that cause proteins to build up in the wrong way. Eventually, this damages cells and causes them to die. It is important to keep a high level of clinical concern when identifying PD because it can be hard to tell it apart from other diseases that are linked to Parkinsonism. Both early- and late-stage difficulties of Parkinson’s disease may currently be addressed with a variety of therapeutic techniques, such as pharmacological treatments and surgical procedures [21,22]. Our proposed approach uses a GNN to track Parkinson’s disease based on the GWT protein-peptide dataset.

## 2. Literature Survey

In this section, we aim to provide a thorough review of previous scientific research and basic ideas in the field of using GNNs for PD. In addition to providing an overview of knowledge, this study will help pinpoint the best ways to build a reference design for GNNs to use in the treatment and prevention of PD [23]. It includes a thorough review of relevant scientific literature and the clarification of important terminology [24]. The following paragraphs provide an overview of the ideas that will be discussed.

Numerous medical investigations, such as those pertaining to PD [25,26], have made extensive use of machine learning (ML) methods, particularly deep learning approaches [27]. In [28], they proposed a model based on deep neural networks to predict how PD will develop. Cognitive outcomes in PD are predicted by Harvey et al. using a combination of various machine learning models. The researchers model the effects of clinical and biofluid predictors. The GenoML open-source Python software version 3.9, developed by [29], incorporates transcriptomics, genetics, and clinical data to provide a peri-diagnostic model for PD risk prediction. The researchers at Park et al. were able to obtain a maximum area under the curve (AUC) of 0.779 [30,31] by using various machine learning algorithms for anthropometric data, laboratory data, and other variables. Based on the illnesses of interest and their associated determinants, the prediction’s effectiveness of existing ML approaches might vary greatly. These techniques range from basic models like decision trees and logistic regression to more complicated ones like deep neural networks. While these ML models perform an excellent task of explaining PD risks, it may be challenging to find the right ML models to use when evaluating PD risk since various PD prediction models use various ML algorithms with distinct sets of predictors. Even though all of the current ML systems for PD risk prediction use the same amount of training data, there has been no effort to systematically compare them. Additionally, research on the key data needed for precise PD risk assessment using each ML technique is lacking. Our research aims to develop the most cutting-edge GWT model, GNN, which will be well-suited for time series prediction using the MDS-UPDRS III prediction scale and will ultimately make clinical evaluations of PD risk easier.

## 3. Materials and Methods

**Figure 1:**
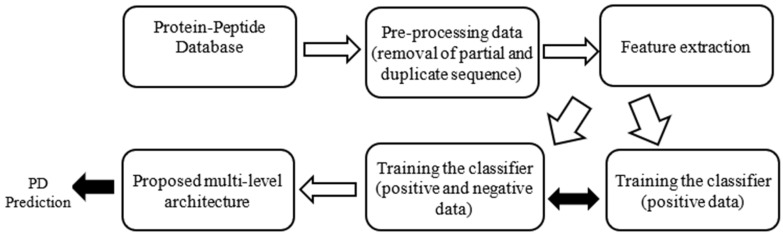
Flow Diagram of Protein–Peptide (PP) Data Based on PD Prediction.

This section explores a complete explanation of each module of the GWT–GNN framework. The method’s process is shown in Figure 1. The suggested GWT–GNN approach improves the model’s performance in predicting the severity of PD symptoms by first determining how similar all PD samples are, then using that information to transform features and obtain more efficient data, and lastly, predicting the severity of PD using the enhanced model. The first step in creating new feature vectors is sorting the features in a weighted graph that is created by GWT. After that, the new attribute representations of the PD data are created by using the GWT to extract various attribute properties of the feature vectors. The next step is to feed the GNN a variety of data attributes in order to take their performance and correlation into full consideration. Finally, the motor-UPDRS-III of PD patients is predicted by combining the weighted findings using the GNN.

Peptides are limited chains of amino acid residues linked together by peptide bonds. They serve several vital activities, including cell signaling and immunological modulation. These molecules mediate from 15% to 40% of all cellular protein-protein interactions [32]. Peptides are a promising class of therapeutic agents because of their structural diversity, adaptability, low resistance, minimal non-target action, and mouldability to engage with certain cellular targets [33]. The past has complicated the search for peptides as medicines due to their short half-life and low oral bioavailability [34,35]. A renewed interest in peptides as potential therapeutic agents has coincided with the development of novel synthetic methods that change their biophysical and biological characteristics [36,37,38,39]. There are now hundreds of peptide medications undergoing clinical trials, and more than 60 have received approval in major pharmaceutical markets [40,41]. Additionally, peptide-like inhibitors have shown promising results in the treatment of autoimmune disorders, cancer, and diabetes in clinical trials [42]. Several next-generation medication candidates have been proposed as potential treatments for type 2 diabetes mellitus. One of them is exenatide, which is a synthetic version of a natural 39-amino acid peptide released by Heloderma suspectum [43].

Pharmaceutical and biotechnological researchers can benefit from a better understanding of protein-peptide complex structure and recognition to create new peptides and peptide-based drugs. The methods of protein-peptide recognition may be better understood by utilizing databases containing protein-peptide complexes. As a result, we train and evaluate the model using this protein-peptide database of PD patients. Figure 1 shows a simplified flow diagram of the dataset process.

**Figure 2:**
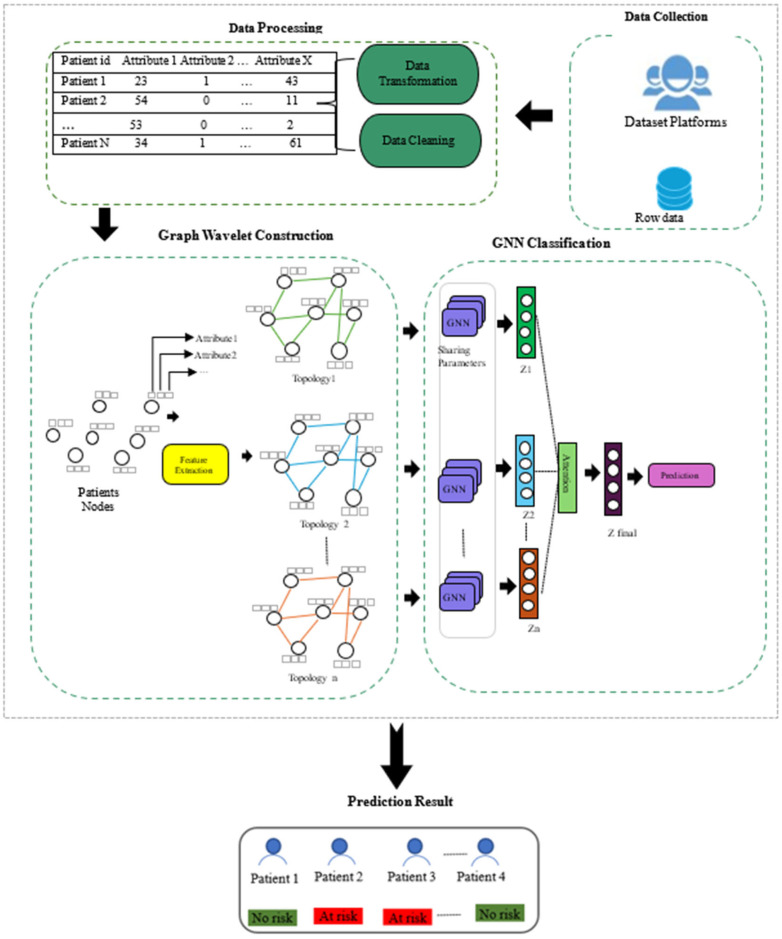
GWT–GNN-based PD Prediction Model.

One of the main goals of the GNN-based PD strategy is to help with PD monitoring by extracting useful insights. Integrating GokWT and heterogeneous data from the PP dataset into a GNN-based architecture is the main focus of this technique. During this preliminary processing phase, activities, including data normalization and standardization, as well as the elimination of unnecessary or irrelevant details, are performed. This requires first standardizing data to make sure it is uniform, then normalizing it such that it is on a consistent scale, and then using noise reduction methods to remove any unnecessary or outlier data. After the GWT time series data prediction is conducted using the given data, the knowledge graph that is created is used to build the best GNN model for PD. The MDS-UPDRS-III is extensively used in this section to assess motor symptoms of PD. Here, we provide a novel approach to PD severity prediction in the graph wavelet domain by use of a Graph Wavelet Transform (GWT). To estimate the severity of patients’ PD time series, the proposed method uses the inverse transform after making predictions of graph wavelet coefficients in the graph wavelet domain. Our method outperforms other approaches, particularly when dealing with non-stationarity or inhomogeneity in the graph data, since it accurately represents the local structure of the distribution of graph data surrounding each patient feature. Consequently, this research set out to determine if a GWT–GNN model could reliably forecast MDS-UPDRS scores from the PP dataset of PD patients. Figure 2 shows the execution of the PD severity prediction on the patient PP dataset.

**Data Pre-processing** 

- Data Cleaning: Improving the quality of data requires cleaning it, which includes deduplicating, patching, correcting, or removing incomplete or inappropriate data.
- Data Transformation: Transferring information from one form to another is known as data transformation. Data transformation, including data size transformation and type transformation, is necessary when the original data type does not match the needs of the model input. Our approach requires numerical inputs to determine data similarity.

### 3.1. Graph Wavelet Transform

**Figure 3:**
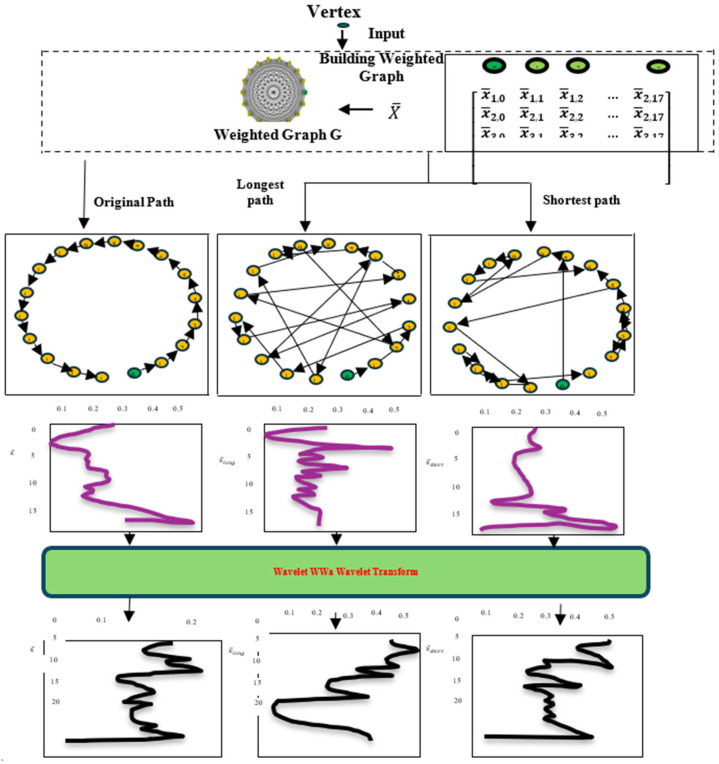
Feature Vector Extraction using Graph Wavelet Transform.

Figure 3 shows the first step of the proposed method, which is to use graph wavelet transform to extract various data features from PD feature vectors. The GWT technique builds a weighted graph G and then uses the wavelet transform to extract vector features, which are represented by various sequences of information. The original PD feature matrix $\bar{X}$ is used to build the G by examining the feature correlations. Hence, when the starting vertex is known, the smoothest and most oscillatory vectors $\bar{x_{min}}$ and $\bar{x_{max}}$, representing the various orders of the vectors, may be obtained from the G using the shortest and longest route search methods, respectively. The PD vector may have several features by using the wavelet transform of three vectors: $\bar{x_{min}}$, $\bar{x_{max}}$ and $\bar{X}$, where X is the original feature vector before sorting to extract features with various frequency scales. Given that $\bar{M}$ is the number of PD patients in the training set and $\bar{Y}$ is the set of all possible values for y, we can say that the PD subset’s training set is $\bar{D}=(\bar{X},\bar{\;Y})={\left\{\left(\bar{x_{i}},\;\bar{y_{i}}\right)\right\}}_{i=1}^{M}$. $\bar{Y}={\left(\bar{y_{1}},\;\bar{y_{2}},\ldots \bar{y_{\bar{M}}}\right)}^{T}$ stands for the MDS-UPDR-III sample’s observed value. $\bar{X}$ is the collection of features from the training set samples, with dimensions $\bar{M}\times N$, and $\bar{x_{i}}$ is the feature vector for the $i^{th}$ sample, with dimension N×1, stated in Equation (1).

(1) $$\bar{X}={\left(\bar{x_{1}},\bar{x_{2}},\ldots ,\bar{x_{\bar{M}}}\right)}^{T}=\left[\begin{array}{c} \begin{array}{ccc} \bar{X_{1,1}} & \bar{X_{1,2}} & \begin{array}{cc} \ldots & \bar{X_{1,N}} \end{array} \end{array}\\ \begin{array}{ccc} \bar{X_{2,1}} & \bar{X_{2,1}} & \begin{array}{cc} \ldots & \bar{X_{2,N}} \end{array} \end{array}\\ \ldots \\ \begin{array}{ccc} \bar{X_{\bar{M},1}} & \bar{X_{\bar{M},2}} & \begin{array}{cc} \ldots & \bar{X_{\bar{M},N}} \end{array} \end{array} \end{array}\right]$$

where $\bar{X_{\bar{M},N}}$ stands for the $N^{th}$ sample feature. The complex nonlinear connection between $\bar{X}$ and $\bar{Y}$ makes it impossible to directly fulfill performance criteria and apply the prediction findings in clinical practice when employing regression algorithms to understand the relationship [44,45]. In order to enhance the accuracy of the predictions, this section employs the GWT approach to derive the new feature $\bar{X}\prime$ from the baseline feature vectors in $\bar{X}$. This feature is then used to determine the mapping connection between $\bar{X}\prime$ and $\bar{Y}$. As a result, the model for making predictions is given in Equation (2):

(2) $${\bar{Y}}^{\prime }=f\left({\bar{X}}^{\prime },\bar{Y}\right)\;$$

#### 3.1.1. Creation of Weighted Graph

Both the target value MDS-UPDRS-III and the inter-PD components of PD are associated. The vector’s frequency characteristics are represented by the sequence information of PD features. The ability of the wavelet transform to extract low-frequency characteristics increases as the data become smoother. In fact, high-frequency characteristics are more easily retrieved when the data are more unstable. The most oscillating and smooth vectors, $\bar{x_{min}}$, $\bar{x_{max}}$, are created by rearranging the features in a weighted graph G that is built based on the correlation between features. This allows for the extraction of various frequency attributes from PD feature vectors.

The formula for the weighted graph is G=(V, E, Dis), where V is the set of N features of PD data $(v_{0},\;v_{1},\;\cdots ,\;v_{N-1}),$ E is the collection of edges between features $\left(v_{0}v_{1},\;v_{1}v_{2},\;\cdots ,\;v_{N-2}v_{N-1}\right)$, and Dis is the weighted adjacency matrix that contains the weights of all relationships. In order to illustrate the relationship between features, we may use Equation (3) to acquire the Euclidean distance $d_{i,j}$ that exists between every feature in $\bar{X}$. The strength of the link increases as the distance decreases. The association weakens as the distance increases.

(3) $$d_{i,j}=\left\{\begin{array}{c} \left\|\bar{x_{:,i}}-\bar{x_{:,j}}\right\|\;if\;i\;\neq j\\ -1\;if\;i=j,\;and\;the\;maximum\;similarity\;is\;searched\\ \infty \;if\;i=j,\;and\;the\;minimum\;similarity\;\;is\;searched \end{array}\right.\;$$

where $x_{:,i}$ denotes the model characteristics of the $i^{th}$ column in X and $x_{:,j}$ stands for the sample features of the $j^{th}$ column. $d_{i,j}$ is a measure of the separation between the $i^{th}$ and $j^{th}$ features in X. The current feature cannot be itself while searching for the most similar (non-similar) data in G; the closest (farthest) feature from it must be identified. Hence, when $i=j,\;d_{i,j}$ is ∞ (−1). This allows us to determine the distance between N features and use Equation (4) to acquire the weighted adjacency matrix Dis in G:

(4) $$Dis=\left[\begin{array}{c} \begin{array}{ccc} d_{1,1} & d_{1,2} & \begin{array}{cc} \ldots & d_{1,N} \end{array} \end{array}\\ \begin{array}{c} \begin{array}{ccc} d_{2,1} & d_{2,2} & \begin{array}{cc} \ldots & d_{2,N} \end{array} \end{array}\\ \begin{array}{c} \ldots \\ \begin{array}{ccc} d_{N,1} & d_{N,2} & \begin{array}{cc} \ldots & d_{N,N} \end{array} \end{array} \end{array} \end{array} \end{array}\right]\;$$

#### 3.1.2. Feature Selection and Wavelet Transform

After obtaining the vectors $\bar{x_{min}}$ and $\bar{x_{max}}$ by organizing the feature sequence along the shortest and longest routes, we inputted $\bar{X}$, $\bar{x_{min}}$, and $\bar{x_{max}}$ into the wavelet transformation in order to extract the associated frequency characteristics from the PD feature vector. In Algorithm 1, we can see the pseudo-code for the method that finds the minimum and maximum similarity data calculation.
**Algorithm 1:** Feature Similarity Calculation using GWT$Input:Weighted\;graph\;G=\left(V,E,Dis\right),where\;V=\left\{v_{0},v_{1},\ldots v_{N-1}\right\},E=\left\{v_{0}v_{1},v_{1}v_{2},\ldots v_{N-2}v_{N-1}\right\}$ $Output:minimum\;and\;maximum\;similar\;feature\;data\;represented\;by\;U_{min}\;and\;U_{max}\;respectively$ Feature Similarity Calculation: 1:For k=0,1,2,…N−1 2:        For minimum similar featurre calculation, while, do $3:\;\;\;\;\;\;\;\;\;\;\;\;Adding\;the\;vertex\;into\;U_{min},\;U_{min}=\{v_{k}\}$ 4:            Continue untill all the data feature path calculation $5:\;\;\;\;\;\;\;\;\;\;\;\;For\;vertex\;v_{k},\;neighbour\;calcualtion\;N\left(k\right)=\left\{v_{j}\in V-U_{min}\;\right|v_{k}v_{j}\in E\}$ $6:\;\;\;\;\;\;\;\;\;\;\;\;Calculate\;v_{j}=argmin\;d_{k,j}=argmin\;\left\|v_{j}-v_{k}\right\|\;w.\;r.\;t.\;v_{j}\in N(k)$ $7:\;\;\;\;\;\;\;\;\;\;\;\;Adding\;the\;vertex\;value\;v_{j}\;to\;the\;end\;of\;the\;U_{min}\;data$ $8:\;\;\;\;\;\;\;\;\;\;\;\;\;Assign\;v_{k}=v_{j}$ $9:\;\;\;\;\;\;\;\;\;\;\;\;Till\;V-U_{min}=\emptyset$ 10:       For maximum similarity calculation, while, do $11:\;\;\;\;\;\;\;\;\;\;\;\;Adding\;the\;vertex\;into\;U_{max},\;U_{max}=\{v_{k}\}$ 12:            Continue untill all the data feature path calculation $13:\;\;\;\;\;\;\;\;\;\;\;\;For\;vertex\;v_{k},\;neighbour\;\;calcualtion\;N\left(k\right)=\left\{v_{j}\in V-U_{max}\;\right|v_{k}v_{j}\in E\}$ $14:\;\;\;\;\;\;\;\;\;\;\;\;Calculate\;v_{j}=argmax\;d_{k,j}=argmax\;\left\|v_{j}-v_{k}\right\|\;w.\;r.\;t.\;v_{j}\in N(k)$ $15:\;\;\;\;\;\;\;\;\;\;\;\;Adding\;the\;vertex\;value\;v_{j}to\;the\;end\;of\;the\;U_{max}\;data$ $16:\;\;\;\;\;\;\;\;\;\;\;\;\;Assign\;v_{k}=v_{j}$ $17:\;\;\;\;\;\;\;\;\;\;\;\;Till\;V-U_{max}=\emptyset$ 18:End

The sorted vector $\bar{x_{min}}$ of PD patients is more uniform, with less high-frequency and more low-frequency components, as compared to the original feature vector X. In contrast, $\bar{x_{max}}\prime s$ high-frequency component grows while its low-level element decreases. By applying the wavelet transform to $\bar{X}$, $\bar{x_{min}}$ and $\bar{x_{max}}$, PD feature vectors may be refined for feature extraction. The characteristics of the vector are wavelet-transformed into its approximation, and detailed components are given in Equation (5):

(5) $$\left\{\begin{array}{c} cA_{1}=\sum_{k}{\bar{x}}_{k}{\bar{H}}_{n-2k}\;;cD_{1}=\sum_{k}{\bar{x}}_{k}{\bar{G}}_{n-2k},\;j=1\\ cA_{j}=\sum_{k}cA_{j-1}{\bar{H}}_{n-2k}\;;cD_{j}=\sum_{k}cD_{j-1}{\bar{G}}_{n-2k},\;j=2,3,\ldots ,J\prime \end{array}\right.$$

where J is the result of the decomposition layer measure, H is the low-pass wavelet filtering coefficient, and G is the high-pass wavelet filtering coefficient. The wavelet coefficients for the $j^{th}$ approximation component $\left(cA_{j}\right)$ and the $j^{th}$ detail component $\left(cD_{j}\right)$ of the signal decomposition are as follows. In order to create a new feature vector representation: $\bar{X}\prime =[cD_{1},\;cD_{2},\cdots ,\;cD_{J},\;cA_{J}],$ the wavelet coefficients of vector x that are generated following wavelet decomposition are combined. The $\bar{x_{min}}\prime$ and $\bar{x_{max}}\prime$ outputs of the wavelet transform on the $\bar{x_{min}}$ and $\bar{x_{max}}$ vectors are also available. Hence, in each subset, X receives three transformation outcomes after feature transformation: $\bar{x}$,$\;\bar{x_{min}}\;$ and $\bar{x_{max}}$. $\bar{X}\prime$ can be defined in the Equation (6):

(6) $${\bar{X}}^{\prime }={\left({\bar{x_{1}}}^{\prime },{\bar{x_{2}}}^{\prime },\ldots {\bar{x_{\bar{M}}}}^{\prime }\right)}^{T}=\left[\begin{array}{c} cD_{1,1}\;cD_{1,2}\ldots \;cD_{1,J}\;cA_{1,J}\\ \begin{array}{c} cD_{2,1}\;cD_{2,2}\ldots cD_{2,J}\;cA_{2,J}\\ \begin{array}{c} \ldots \\ cD_{\bar{M},1}\;cD_{\bar{M},2}\ldots cD_{\bar{M},J}\;cA_{\bar{M},J} \end{array} \end{array} \end{array}\right]\;$$

### 3.2. Graph Neural Network

**Figure 4:**
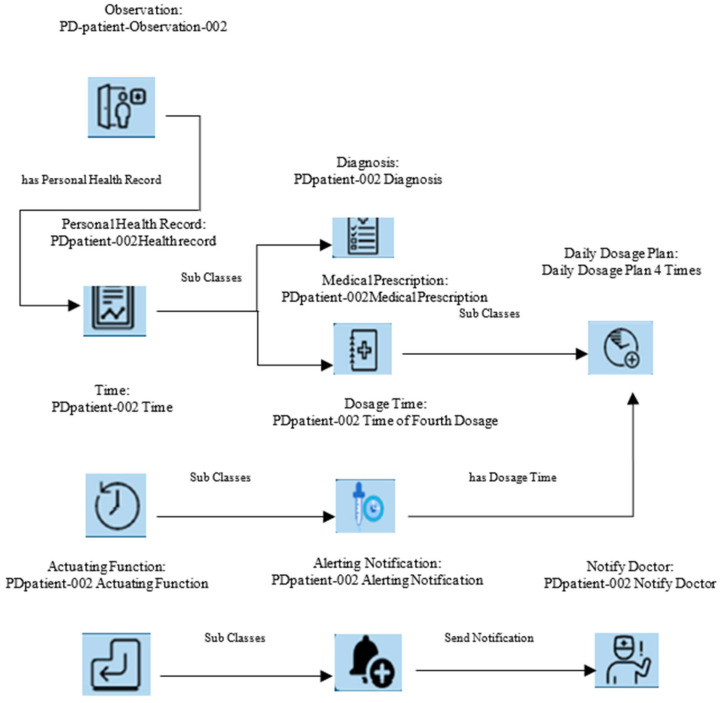
Monitoring PD patients’ Health Report for Severity Prediction.

Graph-structured data are common in PD research, and GNNs are an excellent group of algorithms for dealing with this kind of data [46]. Predictive modelling and pattern recognition are both made possible by these algorithms’ use of graph-based data representation to accurately characterize connections between variables. In order to build a knowledge graph, one must first examine the data in a knowledge base in order to extract triples and then insert them according to a graphical data model. A complete knowledge graph is formed from the characteristics retrieved using GWT via the integration of data triples. In particular, there is a network of interrelationships among these entities due to their interconnection. Figure 4 shows a partial example of a data structure. A medication adherence monitoring and prediction system specifically designed for people with PD may be built upon the conceptual data architecture.

GNN Architecture

In GNN, the three primary levels are the convolutional, max-pooling, and fully connected layers. The result of stacking these layers is a GNN model. On top of these three levels are the dropping layer and the transfer functions. Feature extraction from the input images is the primary focus of the first level of the convolution layers. In order to carry out the statistical convolution operation, each input image is mathematically merged with a convolution filter of a certain size at this level. By dragging the filter over the source images, we can determine the dot product of the filtered and matched input image regions. Feature mapping displays the outcome. Additional levels may use the area of interest as a data source. The parameters that the Conv2D function takes into account are as follows:

(1) The first step in feature extraction is filtering. Different feature detection methods will use different filtering techniques on the input image. The Blur Filters system and the Edge Identification Filters are two of the filters.
(2) The length of the (n × n) convolutional median filter is specified by the kernel length.
(3) Activation—the capacity of a neuron to be triggered. A Rectifier Linear Unit (ReLU) value activates every layer except the output level. They have also added nonlinearity to the system using ReLU. It is essential for detecting linear correlations in feature mapping.
(4) Nodes for Input: This layer has many streams and input images.

GNN Training and Testing Procedure
Training Steps:
Remove the gradient
Completed single forward pass
Determine loss by using nodes for training
Find the gradient and revise the settings as needed.
Testing Steps:
Expected class of nodes
Retrieved class label with the highest possible
Verified the number of accurately predicted values
An accuracy ratio is made by dividing the total number of nodes by the sum of accurate predictions.

The pseudo-code for GNN prediction is shown in Algorithm 2. The method has the option to initialize or accept feature vectors of the graph, edges, and vertices as inputs. We can see the execution being partitioned into layers (line 9). Inside each layer, every edge is simultaneously modified by combining its feature vector with the feature vectors of the related vertices (line 11). By combining the feature vectors of its neighbours with itself, every vertex is simultaneously updated as well (line 15). The combined edges and vertices undergo transformations via combination functions (lines 13 and 17). These functions may take the shape of neural networks. At the end of each iteration, the relevant function, which may be a neural network again, is called upon to provide a readout (line 18).
**Algorithm 2:** GNN for Data Prediction1:Procedure for GNN Prediction 2:      L ←Number of lAyers in GNN 3:      V ←Static Node in graph G 4:      E ←Static Edges in graph G Initialization of all paramaters 5:      for v ∈V do $6:\;\;\;\;\;\;\;\;\;\;\;h_{v}^{0}\;\leftarrow \;\left[x_{v},0,\ldots ,0\right]$ 7:      for e ∈E do $8:\;\;\;\;\;\;\;\;\;\;\;\;g_{e}^{0}\;\leftarrow \;\left[z_{v},0,\ldots ,0\right]$ Layerwise GNN Process 9:      for l=1 to L do Edge Processing:Interchanging the node and edge order 10:           for e ∈E do $11:\;\;\;\;\;\;\;\;\;\;\;\;\;\;\;\;b_{e}^{l}=\rho_{E}^{l}\;\left(\left\{g_{e}^{l-1},h_{v}^{l-1}\;:u\;\in N(e)\right\}\right)$ $12:\;\;\;\;\;\;\;\;\;\;\;\;\;\;\;\;g_{e}^{l}=\emptyset_{E}^{l}\;\left(\left\{b_{e}^{l}\right\}\right)$ Node Processing:Interchanging the node and edge order 13:           for v ∈V do $14:\;\;\;\;\;\;\;\;\;\;\;\;\;\;\;\;a_{v}^{l}=\rho_{V}^{l}\;\left(\left\{h_{v}^{l-1},h_{v}^{l-1}\;:u\;\in N(v)\right\}\right)$ $15:\;\;\;\;\;\;\;\;\;\;\;\;\;\;\;\;h_{v}^{l}=\emptyset_{V}^{l}\;\left(\left\{a_{v}^{l}\right\}\right)$ Readout: $16:\;\;\;\;\;\;\;\;\;\;\;\;\;\;\;\;\hat{y}=\emptyset_{G}\;\left(\rho_{G}\;\left(\left\{h_{v}^{l},g_{e}^{l}\;:v\;,\;e\;\;\in G\right\}\right)\right)$

Assuming any layer l in the interval [1, L], edge transformation takes place as

(7) $$AGGREGATION:b_{e}^{\left(l\right)}=\rho_{E}^{\left(l\right)}\;\left(\left\{g_{e}^{\left(l-1\right)},\;h_{u}^{\left(l-1\right)}:u\;\in \;N(e)\right\}\right)\;\;$$

(8) $$COMBINATION:g_{e}^{\left(l\right)}=\emptyset_{E}^{\left(l\right)}\;\left(\left\{b_{e}^{\left(l\right)}\right\}\right)\;$$

The feature vectors of the vertices at the edge’s endpoints, $h_{u}$, where u ∈ N (e) and the edge’s own feature vector, $g_{e},$ are included in the aggregation of edges, $\rho_{E}$, for the preceding layer l → 1. Based on reference [47], the combination $\phi_{E}$ inputs this the aggregate. The same line of reasoning also applies to the merging and aggregation of nodes, which is given in Equations (7) and (8).

(9) $$AGGREGATION:a_{v}^{\left(l\right)}=\rho_{V}^{\left(l\right)}\;\left(\left\{h_{v}^{\left(l-1\right)},\;h_{u}^{\left(l-1\right)}\;:u\;\in \;N(v)\right\}\right)$$

(10) $$COMBINATION:h_{v}^{\left(l\right)}=\emptyset_{V}^{\left(l\right)}\;\left(\left\{a_{v}^{\left(l\right)}\right\}\right)\;$$

The process of calculating $a_{v}^{\left(l\right)}$, which is the sum of the feature vectors from neighbouring nodes in layer l−1, and the feature vector of layer l, which is determined by using this sum as input $a_{v}^{\left(l\right)}$, are both described by the Equations (9) and (10). Lastly, a readout function is used to acquire the output feature vector $\hat{y}$, which can include combining and aggregating feature vectors from all the edges and vertices in the graph, as well as from the latest iteration of L. This can be given in Equation (11).

(11) $$READOUT:\hat{y}=\emptyset_{G}\;\left(\rho G\left(\left\{h_{v}^{L},g_{e}^{L}\;:v,\;e\;\in \;G\right\}\right)\right)$$

Given that the degree of nodes can vary significantly across a graph, Algorithm 2 assumes that aggregation and combination functions are (i) invariant to permutations of nodes and edges and (ii) invariant to the number of input nodes. This suggests that all edges and vertices can have functions within a layer applied to them simultaneously in any order. Furthermore, if the aggregation function is linear, the sequence of aggregation and combination can be changed. To prevent breaking data dependencies, it is crucial to maintain the layer order, which means that all layer l edge and node actions must be completed before beginning layer l+1 operations.

## 4. Results

### 4.1. Dataset Description and Experimental Setup

This study evaluates the GWT–GNN model’s ability to estimate the severity of Parkinson’s disease (PD) using the public Parkinson’s PP dataset. This dataset has been widely used to estimate the severity of Parkinson’s disease symptoms, and the evaluation results are reliable and accurate. There are three parts to the Protein–Peptide database, each based on a different method: peptide sequences, structure interfaces, and binding sites [48]. The collection is based on protein abundance values from mass spectrometry scans of cerebrospinal fluid (CSF) samples from over a thousand people. Patients with Parkinson’s disease evaluated the severity of the disease and gave multiple samples over a long period of time.

First, pre-processing is employed in the PP dataset to reduce prediction errors in the proposed model. Furthermore, the datasets were randomly split into training and test sets after being randomized to guarantee data integrity. In the experiment, the prediction model’s performance was assessed using 30% of the subset while testing its accuracy and 70% when training the model parameters. Python 3.6 was used to conduct simulations on a Windows 10 system with an Intel Core i5-4590 CPU running at 3.30 GHz.

Furthermore, the mean absolute error (MAE) is computed as follows and serves as the loss function for the GWT–GNN network:

(12) $$MAE=\frac{1}{P}\sum_{i=1}^{P}\left|y_{i}-y_{i}\prime \right|$$

The genuine value is represented by $y_{i}$ and the predicted value is represented by $y_{i}^{\prime }$. In order to assess how well the GWT–GNN model predicts the severity of PD progression and to ensure the accuracy of the predictions, we use MAE and RMSE as the prediction model’s evaluation metrics. Here, Equation (12) is the formula for MAE, and Equation (13) is the formula for RMSE:

(13) $$MSE=\sqrt{\frac{1}{P}\sum_{i=1}^{P}{\left(y_{i}-y_{i}^{\prime }\right)}^{2}\;\;}$$

The accuracy of the prediction model is directly proportional to the degree to which the mean absolute error (MAE) and root-mean-squared error (RMSE) are reduced. The sample features of the PP dataset are illustrated in Table 1.

### 4.2. Experimental Results

An experimental design based on a 70–30 train-test cross validation was used for all procedures. The accuracy and MSE values shown are the averages across all iterations. Table 2 displays the total, motor, and average classification accuracy scores. The findings demonstrate that the GWT–GNN architecture, which simultaneously predicts the score and classifies data, has the greatest performance. When the proposed model is used, the average value, motor, and total are among the best.

**Figure 5:**
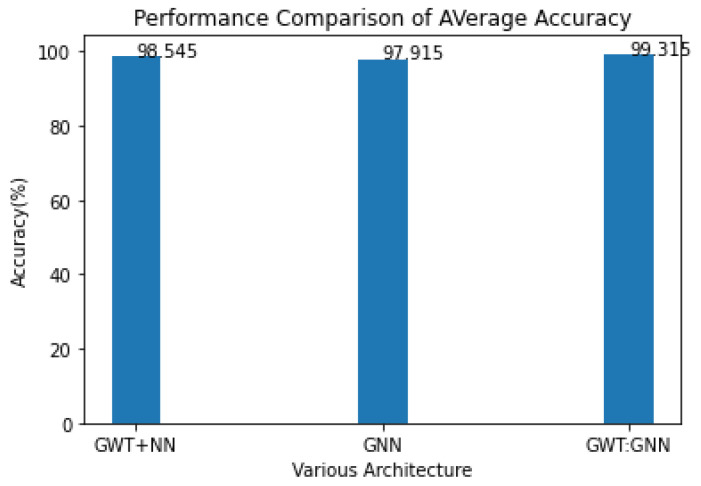
Average accuracy comparison of the proposed model.

The proposed model performance will be evaluated by an ablation study of GWT with NN, GNN, and GWT with GNN using the same PP dataset for score prediction [48]. In Figure 5, we can see the average values for each design, which allows for a more thorough comparison of all the ablation studies. We have already shown that, with a 0.77% improvement in accuracy, the GWT–GNN outperforms all other GNN-based approaches.

**Figure 6:**
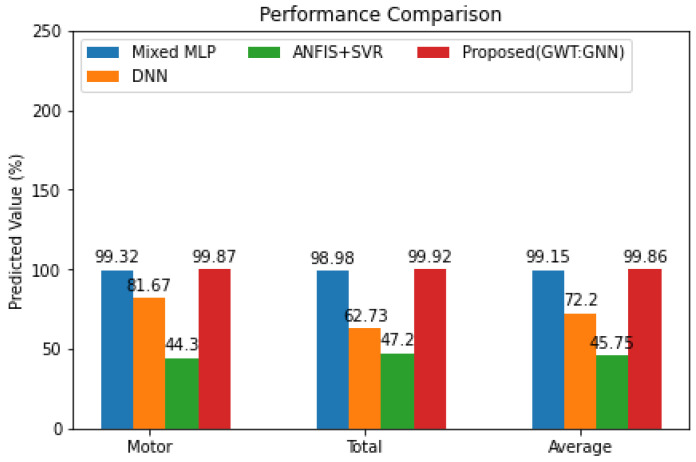
Performance Comparison with State-of-the-Art Methods.

In comparison to the most current State-of-the-Art approaches of mixed MLP [49], ANFIS + SVR [50], and DNN [51], the proposed approach has a higher motor prediction score, as seen in Figure 6. As shown in Figure 6, the motor score, the average score, and the total score are all shown for a variety of models. The explanation for the greatest prediction score is due to the extraction of graphical features for time-based prediction over the PP dataset. When it comes to prediction, the GWT performs quite well on time-based datasets. Once the GWT outputs have been analysed, the weighted features are categorized using the GNN algorithm. As a result, the proposed model had the greatest consecutive prediction score across all three categories.

The Motor and Total UPDRS scores’ corresponding MSE and RMSE values are shown in Table 3 and Table 4, respectively, based on the test dataset. It is clear that the architecture of the proposed model GWT + GNN has provided excellent performance in terms of MSE and RMSE when compared with another ablation model.

Comparisons between the feature extraction module and the Discrete Wavelet Transform (WT), Fourier Transform (FT), and Graph Wavelet Transform (GWT) are shown in Table 5. The findings show that the proposed feature extraction has the lowest mean squared error and root mean squared error in the feature extraction module, likely due to the greater feature relations in the GWT.

**Figure 7:**
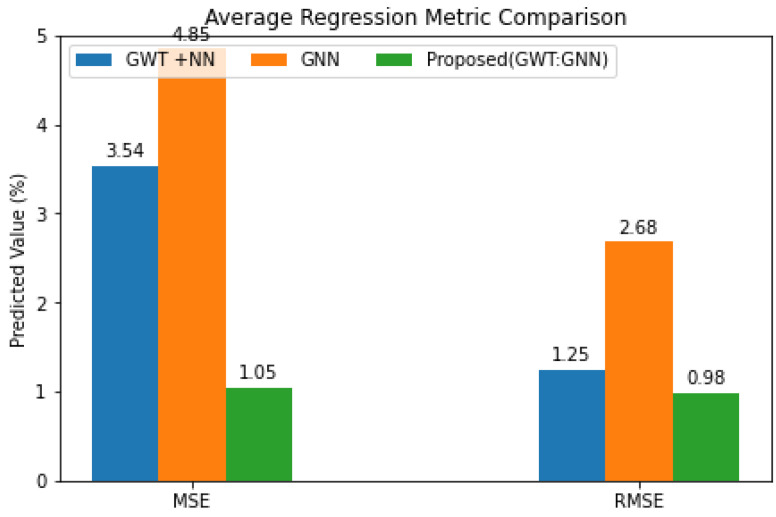
Regression Comparison of Various Architecture of the Proposed Model.

The proposed framework using two regression measures is shown in Figure 7, which is derived from the ablation research. It follows that the proposed GWT–GNN model has the lowest MAE and RMSE. The results demonstrate that, across all categories, the predicted score from the proposed model is in line with the actual score. Although the feature size was lowered by the ablation of GWT with NN, which has a lot of decoders, the prediction was still poor on PP datasets. Similarly, GNN networks’ prediction capabilities would suffer when trained and tested with larger feature datasets. Due to its weighted architecture-based feature extraction, the GWT–GNN hybrid model achieves the highest classification results while minimizing MSE and RMSE.

## 5. Conclusions and Future Work

The purpose of this research was to test the effectiveness of employing a GNN model to predict MDS-UPDRS III scores using the PP dataset of PD patients. Overall, the model performed an excellent task of predicting the MDS-UPDRS part III score, and it performed an adequate task of predicting the axial symptoms subscores as well. According to the results, the model can predict the MDS-UPDRS-III score linked to PD symptoms, including postural abnormalities and gait disruption from the PP dataset. The GWT model is used to analyse and mine the characteristics of the input dataset. Through the use of GWT, the connections and weights of the parameters in the dataset are retrieved. Based on the training characteristics, the features are trained in a GNN model, and then the time series prediction of PD diagnosis is performed. Although there is a need for improvement in the model’s prediction accuracy before it can be used in clinical settings, we see our model as a possible first step in creating a computer-aided approach to analyse PD symptoms using patient PP datasets. The suggested model has a minimal MSE of 0.1796 and an RMSE of 0.2845, according to ablation research. A value of 1.8754 is the MSE when GNN is used alone. It demonstrates that the GWT will choose the most important features to use in the classification model. Data volume, variety, velocity, and integrity will be the focus of future work as we attempt to construct cutting-edge GNN algorithms that are specifically designed to meet the demands of PD research. Developing new methods for representing complex interrelationships in the data is essential to this process.

## Figures and Tables

**Table 1:** Features of PP Datasets.

S.NO	Features
1	PDB
2	Protein Name
3	Resolution
4	Classification
5	Peptide Chain
6	Peptide Size
7	Peptide Sequence
8	Peptide Description
9	Peptide Organism
10	Peptide Interface Area
11	Peptide Molecular Weight
12	Peptide Aromaticity
13	Peptide Instability
14	Peptide Isoelectric Point
15	Receptor Chain
16	Receptor Size
17	Receptor Sequence
18	Receptor Description
19	Receptor Organism
20	Receptor Interface Area
21	Receptor Molecular Weight
22	Receptor Aromaticity
23	Receptor Instability
24	Receptor Isoelectric Point
25	Sequence Cluster
26	Is Sequence Cluster Centroid
27	Interface Cluster
28	Is Interface Cluster Centroid
29	Binding Cluster
30	Is Binding Cluster Centroid

**Table 2:** Performance Comparison of Ablation Study.

Net	Motor (%)	Total (%)	Average (%)
GWT + NN	98.41	98.68	98.545
GNN	97.26	98.57	97.915
GWT + GNN	99.79	98.84	99.315

**Table 3:** Regression results for motor scores.

Net	MSE	RMSE
GWT + NN	0.98542	0.9584
GNN	1.8542	1.3846
GWT + GNN	0.1458	0.2547

**Table 4:** Regression results for total score.

Net	MSE	RMSE
GWT + NN	0.8657	0.8864
GNN	1.8754	2.5842
GWT + GNN	0.1796	0.2845

**Table 5:** Performance Comparison on Feature Extraction.

Net	MSE	RMSE
DWT + GNN	0.2845	0.3572
FT + GNN	0.5487	0.4685
GWT + GNN	0.1796	0.2845

## Data Availability

Data will be made available on request.
